# Breast cancer–the impact of conflict and displacement

**DOI:** 10.1016/j.eclinm.2024.102906

**Published:** 2024-10-24

**Authors:** eClinicalMedicine

October marks global breast cancer awareness month. Breast cancer accounts for 11.6% of all cancers. Despite being the most common type of female cancer, breast cancer awareness levels vary globally. Self-examination, education, and breast cancer screening are imperative for early detection, treatment, and preservation of life.

Many high-income countries provide nationwide screening programmes. Countries such as the UK offer screening every 3 years for women aged 50 years and older, and the USA every 2 years from the age of 40 years. In contrast, many low-income and middle-income countries frequently encounter increased challenges and struggle to establish and maintain robust screening programmes. Barriers to screening in lower income countries are associated with poor funding, and inadequate health education programmes. Shortages in access to health-care facilities and diagnostic equipment in rural settings and areas affected by conflict, also affect the quality of care. With over 100 ongoing armed conflicts globally, humanitarian crises often lead to mass disruption of care systems and the displacement of individuals, resulting in hidden populations of unscreened and untreated patients.

Recent studies of refugee populations in Türkiye and Jordan, highlight the incidence of late-stage breast cancer, poor access to treatment, and low breast cancer awareness. Studies found that 82 (82%) of 100 Afghan refugees in Türkiye were unaware of the warning signs associated with breast cancer, while 102 (87.2%) of 117 Syrian refugees, over the age of 25 years, had never undergone a breast examination. It is therefore unsurprising that breast cancer was found to be one of the most common cancers experienced by Syrian refugees (148 [30%] of 493 women), with late-stage detection and disrupted care leading to an estimated survival rate of 37.8%.

The ongoing war in Ukraine is also predicted to impact cancer diagnosis. With 90% of Ukrainian refugees thought to be women, disruption to care will likely supersede the nationwide early screening programmes, put in place in 2020, to combat already low breast cancer screening uptake. To alleviate impact, neighbouring countries such as Poland, Czech Republic, and Romania have pledged to absorb the burden of cancer care for Ukrainian refugees. Similarly, the ongoing destruction of medical infrastructure and cancer facilities in Gaza, present additional challenges for the delivery of care. The impact such crises have over access to care is difficult to measure as conflicts persist. However, with a 99% survival rate when detected at an early localised stage, delays in breast cancer diagnosis and treatment due to conflict, will continue to prove detrimental.

At the end of 2023, the Global Summit on War and Cancer focused attention on non-communicable diseases and cancer care. In response, the European Cancer Organisation (ESO), UN, and WHO launched their joint manifesto addressing the devastating impact of conflict on cancer care. The overarching goal is to embed cancer care as a mainstay in emergency response plans in all conflict settings. They aim to establish working groups, generate online coordination platforms, and develop action plans supporting access, provision, research, and care. The manifesto also demands that the Geneva Convention is fully upheld to protect medical workforce and patients; prohibiting attacks on medical facilities. Many conflicts and recent strikes, regrettably undermine these primary objectives. Towards the end of 2023, the only cancer hospital in Gaza was lost to the conflict; while airstrikes in Lebanon, Ukraine, and many other zones pose continued threat to medical facilities and personnel.

Rapid destruction of health-care infrastructure, displacement of medical professionals and declining access to medical care mean that the true extent of cancer, and breast cancer incidence, will remain difficult to measure for years to come. As a leading cause of cancer mortality, many host countries are addressing ways to mitigate the impact of conflict on screening. Some host countries, such as Türkiye and Moldova, have introduced free or low-cost screening for Ukrainian refugees and are developing telehealth support and treatment programmes for registered refugees. While both the UK and the Netherlands are working to integrate registered refugees into national health-care databases, automatically enrolling participants into screening programmes for breast cancer.

Language, culture, faith, and unfamiliarity with a country’s health-care system, are some of the complex challenges refugees face when accessing breast cancer screening and care. To support engagement efforts, the US Centres for Disease Control promotes breast screening 30–90 days following arrival as part of routine medical examination in women older than 40 years. In Australia, The New South Wales Government has also introduced a Refugee Health Flexible Fund, which is promoted through community champion training, in-language media articles, and resource development.

While some countries rise to the challenges posed by conflict, action points laid out by the ESO–UN–WHO manifesto underline the importance of cancer services in refugee supporting countries and fragile conflict zones. Through inclusive strategies and models, improved access to care providers, translation services, online coordination platforms, and educational resources, refugee access to breast cancer screening and treatment facilities will be strengthened. As awareness efforts continue to improve, the care of non-communicable diseases, such as breast cancer, in conflict zones will hopefully become a mainstay of humanitarian support.

